# Diagnostic performance evaluation of different TI-RADS using ultrasound computer-aided diagnosis of thyroid nodules: An experience with adjusted settings

**DOI:** 10.1371/journal.pone.0245617

**Published:** 2021-01-15

**Authors:** Nonhlanhla Chambara, Shirley Y. W. Liu, Xina Lo, Michael Ying

**Affiliations:** 1 Department of Health Technology and Informatics, The Hong Kong Polytechnic University, Hung Hom, Kowloon, Hong Kong, SAR, China; 2 Department of Surgery, Prince of Wales Hospital, Shatin, New Territories, Hong Kong, SAR, China; 3 Department of Surgery, North District Hospital, Sheung Shui, New Territories, Hong Kong SAR, China; Taipei Medical University, TAIWAN

## Abstract

**Background:**

Thyroid cancer diagnosis has evolved to include computer-aided diagnosis (CAD) approaches to overcome the limitations of human ultrasound feature assessment. This study aimed to evaluate the diagnostic performance of a CAD system in thyroid nodule differentiation using varied settings.

**Methods:**

Ultrasound images of 205 thyroid nodules from 198 patients were analysed in this retrospective study. AmCAD-UT software was used at default settings and 3 adjusted settings to diagnose the nodules. Six risk-stratification systems in the software were used to classify the thyroid nodules: The American Thyroid Association (ATA), American College of Radiology Thyroid Imaging, Reporting, and Data System (ACR-TIRADS), British Thyroid Association (BTA), European Union (EU-TIRADS), Kwak (2011) and the Korean Society of Thyroid Radiology (KSThR). The diagnostic performance of CAD was determined relative to the histopathology and/or cytology diagnosis of each nodule.

**Results:**

At the default setting, EU-TIRADS yielded the highest sensitivity, 82.6% and lowest specificity, 42.1% while the ATA-TIRADS yielded the highest specificity, 66.4%. Kwak had the highest AUROC (0.74) which was comparable to that of ACR, ATA, and KSThR TIRADS (0.72, 0.73, and 0.70 respectively). At a hyperechoic foci setting of 3.5 with other settings at median values; ATA had the best-balanced sensitivity, specificity and good AUROC (70.4%; 67.3% and 0.71 respectively).

**Conclusion:**

The default setting achieved the best diagnostic performance with all TIRADS and was best for maximizing the sensitivity of EU-TIRADS. Adjusting the settings by only reducing the sensitivity to echogenic foci may be most helpful for improving specificity with minimal change in sensitivity.

## Introduction

Thyroid cancer is the most common endocrine malignancy which constitutes about 5% of all cancers [1, 2]. With the advancement and increased sensitivity of diagnostic imaging tools such as ultrasound, the incidence of thyroid cancers is rising particularly for subclinical cases [3]. Ultrasound-guided fine-needle aspiration cytology (FNAC) is the reference standard pre-operatively; however, its major drawback is the indeterminate results category which has about 25% possibility of malignancy [4]. Although ultrasound is the recommended primary imaging modality for thyroid nodule assessment, it has drawbacks of being operator-dependent and the subjective interpretation of results. Various thyroid malignancy risk classification guidelines have been designed to assist with categorizing risk of malignancy based on several predictive sonographic features. Some of the commonly used guidelines are the American College of Radiology (ACR) Thyroid Imaging Reporting and Data System (TI-RADS) [5], American Thyroid Association (ATA) [6], British Thyroid Association (BTA) [7] the Korean Society of Thyroid Radiology (KSThR) [8], Kwak- TI-RADS [9], and the European Thyroid Association (EU-TIRADS) [10]. The diversity of sonographic features highly predictive of malignancy in the different guidelines augments the dependence on the experience and clinical approach of the clinician [11].

Computer-aided diagnosis (CAD) systems have been proposed to offer a more objective and consistent interpretation of sonographic features in comparison with human visual assessment due to their computational analysis of sonographic textural features [12, 13]. Recent studies have shown that thyroid CAD systems have a diagnostic performance that is comparable to that of experienced radiologists with combined techniques having more potential for superior performance [14, 15]. One globally approved thyroid ultrasound CAD system that allows for simultaneous diagnosis of thyroid nodules with different TIRADS is AmCAD-UT (AmCad Biomed, Taipei, Taiwan). This CAD software has been evaluated for diagnostic performance in differentiating malignant and benign thyroid nodules in a few studies. Some studies demonstrated its comparable sensitivity in comparison with clinical experts and radiologists and its role in improving sonographers’ interpretations of space-occupying thyroid lesions [16, 17]. Although AmCAD-UT settings can be adjusted for optimised diagnostic performance, these previous studies only assessed the diagnostic performance using the default settings with a very limited comparison of multiple TIRADS.

The aim of the present study was to evaluate the diagnostic performance of AmCAD-UT at varied detection sensitivity settings of different ultrasound features for thyroid nodule differentiation based on six different TIRADS within the software. To the best of our knowledge, the value of adjusting AmCAD-UT settings in comparison with the default setting for thyroid nodule assessment has not yet been explored.

## Materials and methods

### Study type and data sources

This retrospective study was approved by the Human Subjects Ethics Subcommittee of The Hong Kong Polytechnic University (Registration Number: HSEARS20190123004). A consecutive case analysis approach was used for the data collection of thyroid nodule ultrasound images. Due to the retrospective nature of this study individual informed consent was waived.

Images of thyroid nodules were obtained from image archives of thyroid ultrasound studies previously conducted by our research group and from an open access thyroid ultrasound image database, Digital Database of Thyroid Ultrasound Images (DDTI) (Universidad Nacional de Colombia, CIM@LAB and Instituto de Diagnostico Medico (IDIME), Bogota, Colombia) [18]. Images from the previous studies by our research group were all acquired using a Supersonic Aixplorer ultrasound machine (SuperSonic Imagine, Aix-en-Provence, France) and a 4–15 MHz linear transducer. These images have not been used in any grey-scale ultrasound CAD analysis study before. For the images obtained from the online database, details of the types of ultrasound machines used were a TOSHIBA Nemio 30 and a TOSHIBA Nemio MX (Canon Medical Systems, Tochigi, Japan) with 12 MHz linear and convex transducers [18].

### Image selection criteria

**Fig 1:**
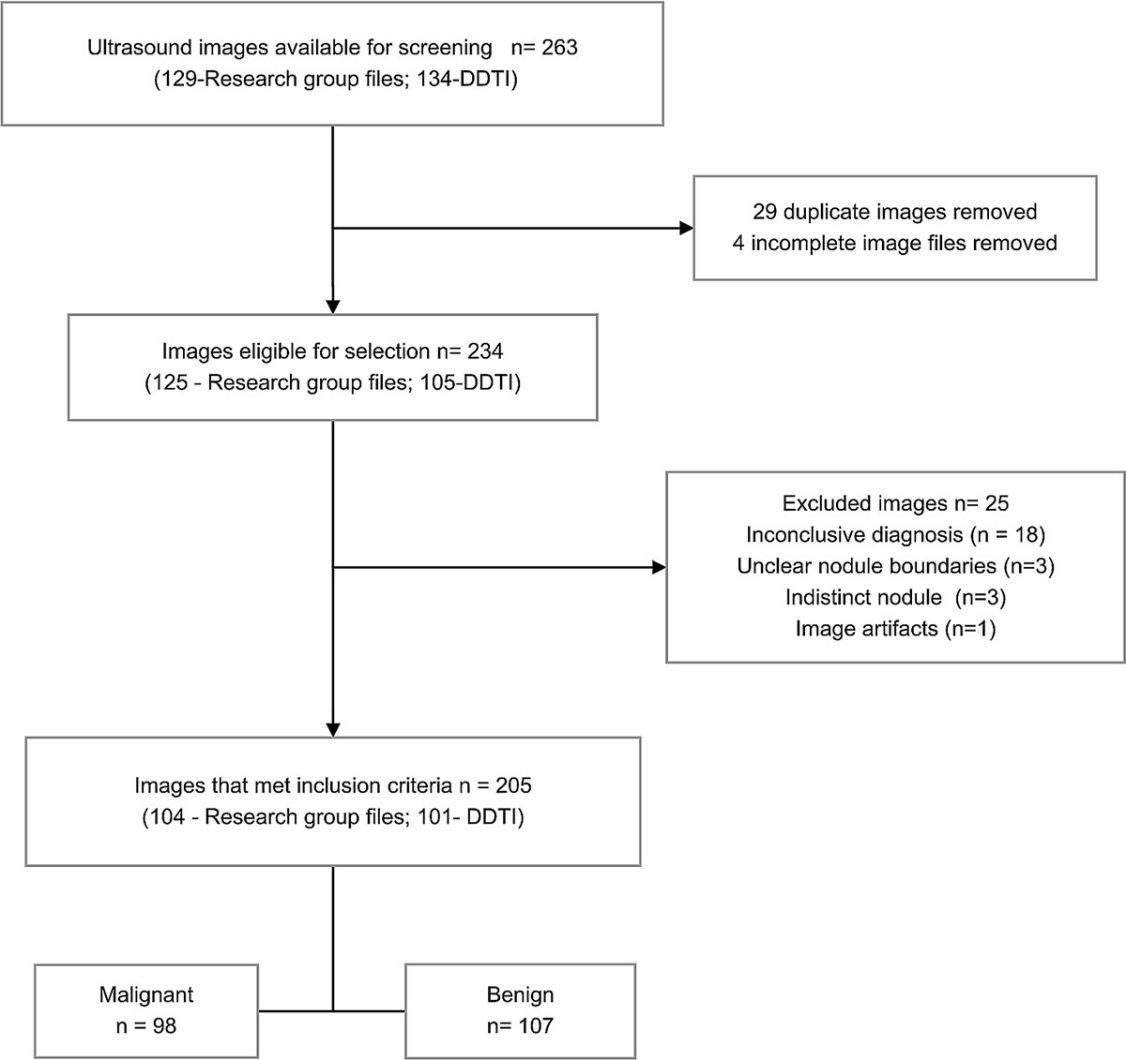
A flowchart illustrating the ultrasound image selection procedure.

A sonographer with more than 15 years’ experience in thyroid ultrasound reviewed the ultrasound images individually. 263 images from 198 patients (94- DDTI; 104-research group files) were retrieved from both thyroid ultrasound image sources for the initial selection process. 129 of these were from our previous research group studies done between February 2013 and December 2014, and 134 images were from the DDTI database. The inclusion criteria were diagnostically acceptable thyroid nodule B-mode ultrasound images from adult patients with thyroid cancer suspicion and confirmatory cytological and/or histopathological results. Incomplete ultrasound images without clear boundaries, indeterminate cytology and/or no histopathology results were excluded from the study. Two thyroid surgeons with extensive experience conducted the fine-needle aspiration cytology of thyroid nodules and provided cytological and histopathological results. Images from the DDTI database had a cytological diagnosis as had been determined by experts [18]. The standard of reference that was used to differentiate benign and malignant nodules was a cytological diagnosis and/or histopathology results. A total of 205 images (104-research group files; 101-DDTI files) met the inclusion criteria and were evaluated in this study. Fig 1 shows the ultrasound image selection process.

Areas clearly demonstrating the nodule were selected and separated from the entire image and the new nodule-specific images were coded and saved in JPEG format.

### CAD analysis of the thyroid nodule images

A radiographer with 2-years’ thyroid ultrasound experience performed the CAD analysis using the AmCAD-UT thyroid CAD software after a month of training in using the software. The user was blinded to the cytology and/or histopathology results.

#### CAD ROI-selection

The coded JPEG images were uploaded onto the AmCAD-UT software user interface for analysis. From the 3 methods of selecting the region of interest (ROI) within the software; manual outlining was adopted for this study as it ensured a standardized approach than the semi-automated and the automated nodule recognition methods, which missed some nodule areas during the training period. After selecting the ROI, the user then adjusted the different settings for ultrasound feature analysis before confirming the analysis for the diagnosis output.

#### CAD settings selection

The AmCAD-UT software can be adjusted for detection sensitivity within pre-determined ranges for margins (1–5), hyperechoic foci (2.0–4.0) and anechoic areas (0–0.5), while visualization can be modified for echogenicity (-50–50) and texture (10–100). Detection sensitivity increases with an increase in the settings for the different ultrasound features except for hyperechoic foci setting which has an inverse relationship. The standalone diagnostic performance of AmCAD-UT established at its development phase testing showed that the detection of “hyperechoic foci” is dependent on detected “anechoic areas”. The highest diagnostic performance of over 90% was achieved at a hyperechoic setting of 3.5 for different ranges of “anechoic areas”, with comparable high performance at 0.2 and 0.5 settings; whereas margins had the best performance using the 2.0 and 3.0 setting based on images from 3 different ultrasound machines [19]. The commonly used default setting uses median values for all the parameter settings. Based on this background, this present study sought to determine the setting for optimised diagnostic performance between the default settings and the “hyperechoic foci” maintained at 3.5 with variations of “anechoic areas” and “margins” at settings that previously achieved the highest diagnostic performance during the development phase testing. “Echogenicity” and “texture” parameter settings were consistently maintained at median values for the objective comparative analyses with the default setting. These two parameter settings mainly influenced subjective visualization of the images without a change in CAD diagnosis output during our pilot testing of the software. The different sonographic settings used for the comparisons in this study are tabulated in Table 1.

**Table 1:** Different AmCAD-UT settings adjustment for comparative analysis of diagnostic performance.

Name of setting	Anechoic area	Hyperechoic foci	Margin	Echogenicity	Texture
**Default**	0.2	2.8	3.0	0	33
**Adjusted 1**	0.2	3.5	3.0	0	33
**Adjusted 2**	0.5	3.5	2.0	0	33
**Adjusted 3**	0.5	2.8	2.0	0	33

#### CAD TI-RADS output

**Fig 2:**
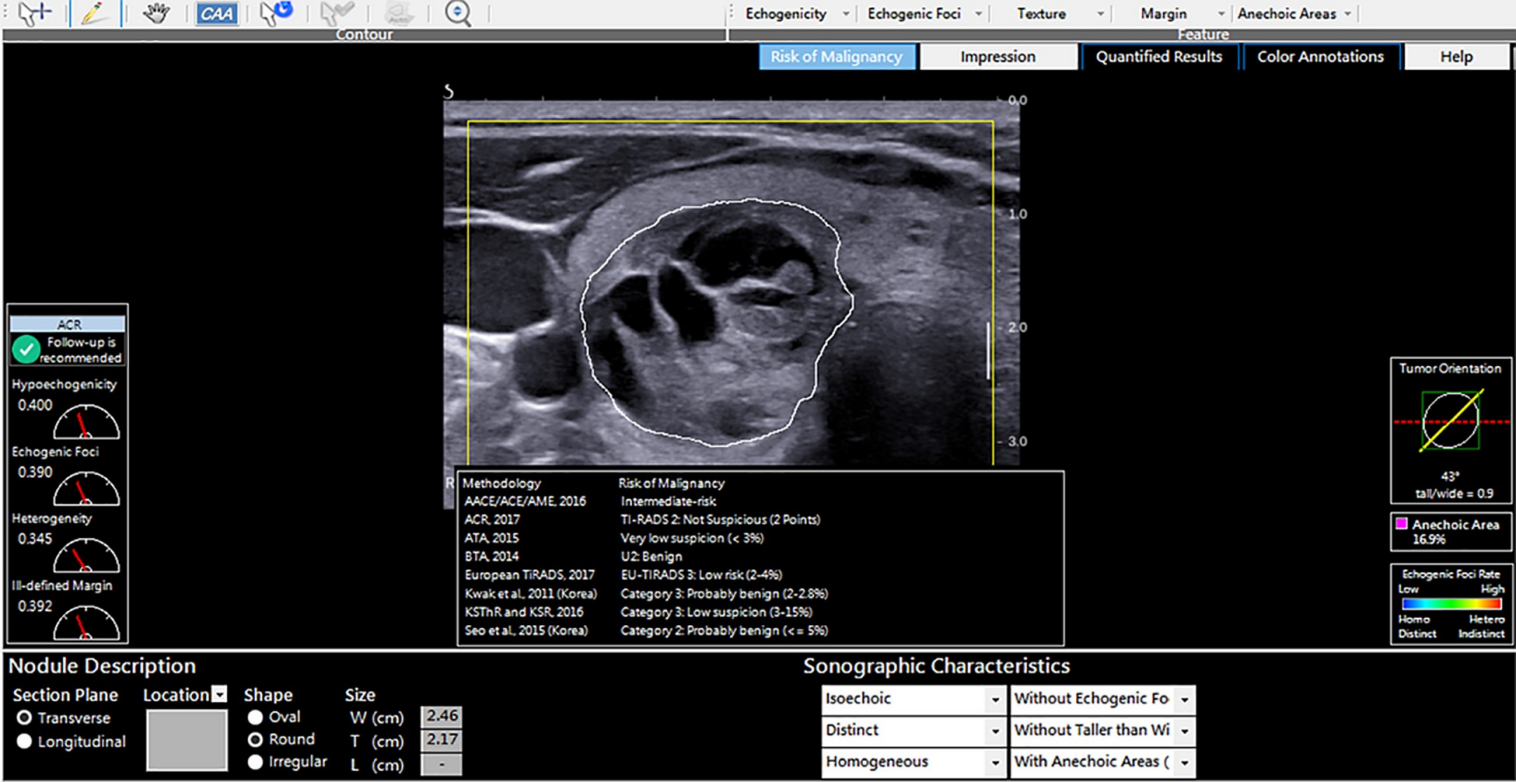
AmCAD-UT diagnosis output from different TIRADS for a cytologically benign nodule.

The software computed the malignancy risk category of the nodules based on 8 malignancy risk stratification systems in the software as demonstrated in Fig 2. To determine the diagnostic performance of the CAD software, the CAD risk stratification output for each nodule based on 6 risk stratification systems (ACR; ATA, BTA, EU, Kwak and KSThR TIRADS) was compared to the ground truth which was the final cytological or histopathological diagnosis. AACE/ACE/AME and Seo et al., 2015 TIRADS were excluded from the analysis as this study evaluated TIRADS with 5 or more risk stratification categories.

### Data analysis and statistical analysis

The statistical analysis was performed using the SPSS software package (version 26.0, SPSS Inc., Chicago, IL, USA). Categorical variables were expressed as percentages and continuous variables were expressed as mean values ± standard deviation. The diagnostic performance measures: sensitivity (SEN), specificity (SPEC), negative predictive value (NPV), positive predictive value (PPV), diagnostic accuracy (DA) and corresponding 95% confidence intervals (CI) were calculated and the Cochran’s Q test and McNemar test were used for comparative analysis of the risk stratification systems. Results with the Bonferroni correction for multiple comparisons were adopted for the Cochran’s Q-test. The receiver operating characteristic (ROC) curves were generated and the areas under the ROC curve (AUROC) were calculated to determine the diagnostic performance of AmCAD-UT for the 6 risk stratification systems and the z-test was used to compare the AUROC of different TIRADS. Precision-recall curves were also generated to complement the ROC results [20]. The optimal cut-off points were obtained from the ROC curves and a cut-off point that resulted in a compromise of both sensitivity and specificity with the least difference between the two at a higher sensitivity was deemed optimal [21, 22]. The tests were two-sided and P< 0.05 denoted statistical significance.

## Results

### Characteristics of the thyroid nodules

This study included 205 thyroid nodules comprising of 98 (48%) malignant nodules and 107 (52%) benign nodules from 198 patients (170 females; 28 males). The age range of the patients was 75 years (21–95) with a mean age of 53.4 years ± 14.7. The mean short-axis diameter for malignant nodules was 1.75 ± 0.93cm (range 0.08–4.29) while that of benign nodules was 2.64 ± 1.70cm (range 0.24–8.74). Thirty-five nodules (13 benign and 22 malignant) had a diameter less than 1cm. There were 77 nodules sized between 1 and 2cm (31 benign and 46 malignant) while 93 nodules were greater than 2cm (63 benign and 30 malignant).

### Diagnostic performance of AmCAD-UT at different adjusted settings

**Fig 3:**
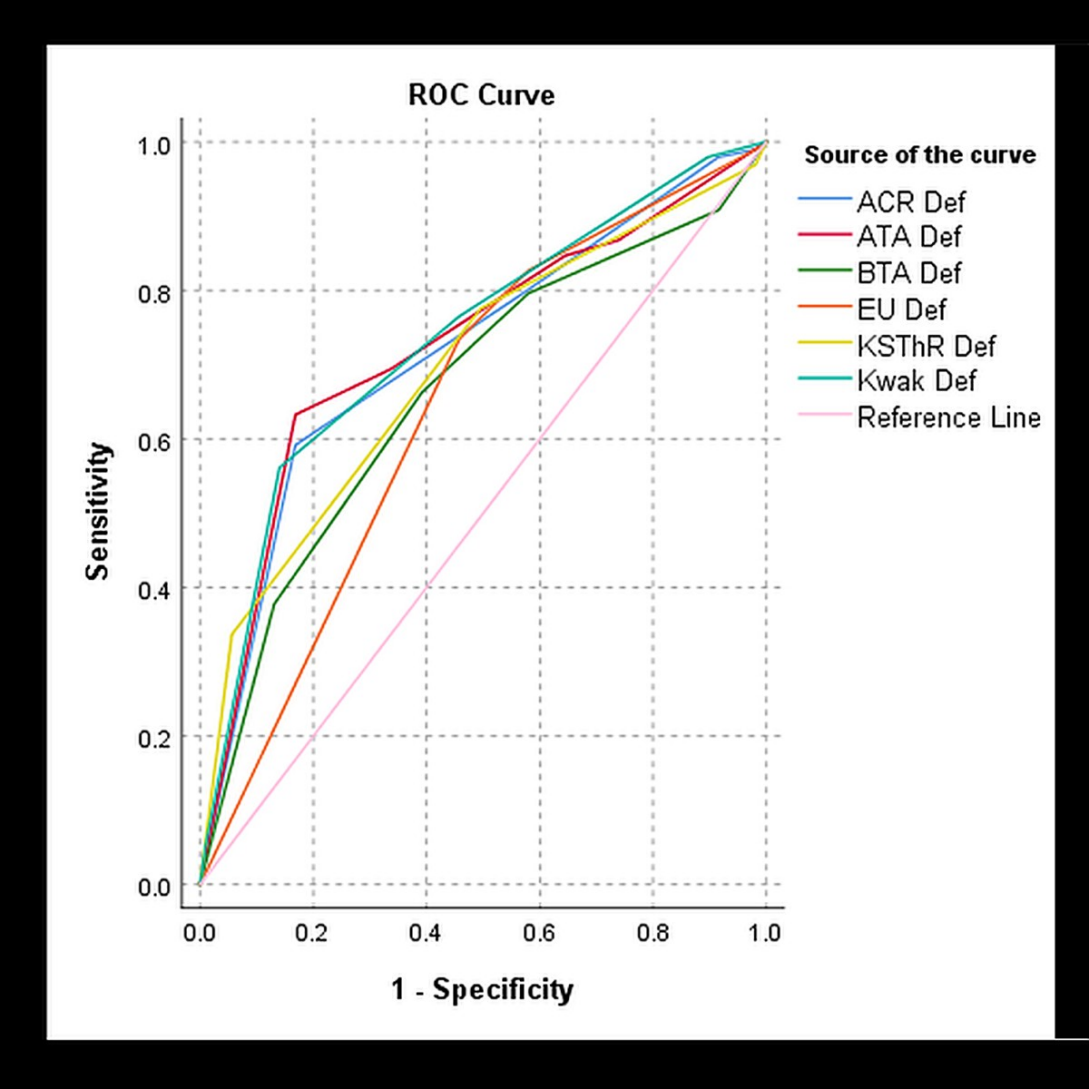
ROC curve demonstrating the diagnostic performance of the 6 TIRADS at the default setting.

**Fig 4:**
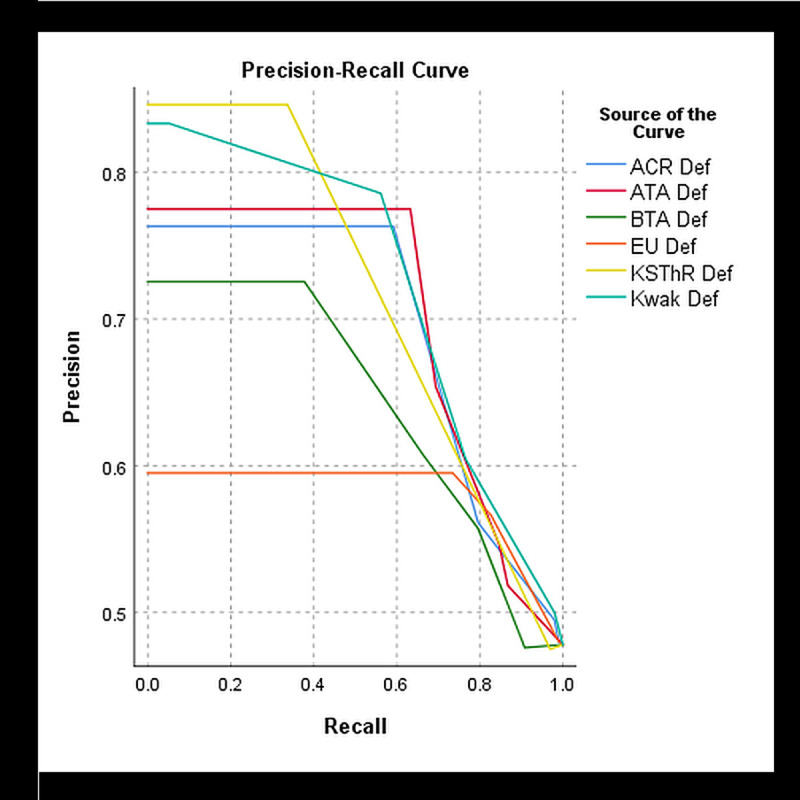
PRC curve demonstrating the diagnostic performance of the 6 TIRADS at the default setting.

The diagnostic performance measures for the 205 nodules were analysed at the different adjusted settings. Table 2 shows the results. The optimal TIRADS cut-off point was determined to be category 4 which was the moderate suspicion with ACR, intermediate suspicion with ATA, Kwak, EU and KSThR and suspicious level with BTA TIRADS. The best optimal diagnostic performance for all diagnostic measures was achieved at the default setting with all TIRADS. At this setting, Kwak TIRADS had the highest AUROC (0.74). EU TIRADS achieved the highest sensitivity and NPV and lowest specificity (SEN: 82.7%, NPV: 72.6%, SPEC: 42.1%). ATA TIRADS had the highest specificity and PPV (SPEC: 67.3%, PPV: 65.4%). BTA TIRADS yielded the lowest sensitivity and NPV (SEN: 66.3%, NPV: 66.3%) while ACR TIRADS has the lowest PPV (56.1%). The six TIRADS generally had similar diagnostic performance and discrimination of benign and thyroid nodules as illustrated by the ROC curve (Fig 3). ACR, ATA, Kwak and KSThR TIRADS had good diagnostic accuracy based on the AUROC of 70% and above. At the chosen TIRADS cut-off category, the optimal precision and recall were derived from Kwak and ATA TIRADS (Fig 4). All TIRADS generally had high precision at low recall at different cut-off points such that even at the optimal cut-off category the PPV was substantially lower than the sensitivity.

**Table 2:** AmCAD-UT Diagnostic performance at different adjusted settings.

AmCAD-UT Setting	TIRADS	SEN % (CI)	SPE (%) (CI)	^ᵻ^PPV (%) (CI)	^ᵻ^NPV% (CI)	^ᵻ^DA (%) (CI)	AUROC (CI)
**Default**	ACR-4	79.6 (70.3; 87.1)	43.0 (33.5; 52.9)	56.1 (51.3; 60.8)	69.7 (59.5; 78.3)	60.5 (53.4; 67.2)	.72 (.65; .79)
	ATA-4	69.4 (59.3; 78.3)	66.4 (56.6; 75.2)	65.4 (58.4; 71.8)	70.3 (63.1; 76.7)	67.8 60.9; 74.1)	.73 (.66; .80)
	BTA-4	66.3 (56.1; 75.6)	60.8 (50.8; 70.1)	60.8 (54.0; 67.1)	66.3 (58.9; 73.0)	63.4 (56.4; 70.0)	.66 (.59; .74)
	EU-4	82.7 (73.7; 89.6)	42.1 (32.6; 52.0)	56.6(52.1; 61.1)	72.6 (62.0; 81.2)	61.5 (54.4 to 68.2)	.65 (.58; .71)
	Kwak-4b	76.5 (66.9; 84.5)	54.2 (44.3; 63.9)	60.5 (54.8; 65.9)	71.6 (62.9; 79.0)	64.5 (57.9; 71.4)	.74 (.67; .80)
	KSThR-4	77.6 (68.0; 85.4)	50.5 (40.6; 60.3)	58.9 (53.5; 64.1)	71.1 (61.9; 78.8)	63.4 (56.4; 70.0)	.70 (.63; .76)
**Adjusted1**	ACR-4	74.5 (64.7; 82.8)	48.6 (38.8; 58.5)	57.0 (51.6; 62.3)	67.5 (58.5; 75.5)	61.0 (53.9; 67.7)	.67 (.60; .74)
	ATA-4	70.4 (60.3; 79.2)	67.3 (57.6; 76.1)	66.4 (59.3; 72.7)	71.3 (64.0; 77.6)	68.8 (62.0; 75.1)	.71 (.64; .78)
	BTA-4	69.4 (59.3; 78.3)	58.9 (49.9; 68.3)	60.7 (54.3; 66.8)	67.7 (60.0; 74.6)	63.9 (56.9; 70.5)	.67 (.59; .74)
	EU-4	81.6 (72.5; 88.7)	46.7 (37.0; 56.6)	58.4 (53.5; 63.2)	73.5 (63.6; 81.5)	63.4 (56.4; 70.0)	.66 (.59; .73)
	Kwak-4b	71.4 (61.4; 80.1)	57.0 (47.1; 66.5)	60.3 (54.2; 66.2)	68.5 (60.5; 75.6)	63.9 (56.9; 70.5)	.68 (.61; .75)
	KSThR-4	71.4 (61.4; 80.1)	58.9 (49.0; 68.3)	61.4 (55.1; 67.3)	69.2 (61.3; 76.2)	64.9 (57.9; 71.4)	.70 (.63; .76)
**Adjusted2**	ACR-4	61.2 (50.9; 70.9)	57.9 (48.0; 67.4)	57.1 (50.4; 63.7)	62.0 (54.8; 68.7)	59.5 (52.5; 66.3)	.62 (.55; .70)
	ATA-4	44.9 (34.8; 55.3)	78.5 (69.5; 85.9)	65.7 (55.6; 74.5)	60.9 (55.9; 65.6)	62.4 (55.4; 69.1)	.59 (.51; .67)
	BTA-4	56.1 (45.7; 66.1)	64.5 (54.7; 73.5)	59.1 (51.5; 66.4)	61.6 (55.2; 67.6)	60.5 (53.4; 67.2)	.60 (.53; .68)
	EU-4	71.4 (61.4; 80.1)	55.1 (45.2; 64.8)	59.3 (53.3; 65.1)	67.8 (59.6; 75.1)	62.9 (55.9; 69.6)	.65 (.57; .72)
	Kwak-4b	50.0 (39.7; 60.3)	68.2 (58.5; 76.9)	59.0 (50.6; 67.0)	59.8 (54.1; 65.4)	59.5 (52.5; 66.3)	.61 (.54; .68)
	KSThR-4	59.2 (48.8; 69.0)	62.6 (52.7; 71.8)	59.2 (51.9; 66.1)	62.6 (55.9; 68.9)	61.0 (53.9; 67.7)	.62 (.55; .69)
**Adjusted3**	ACR-4	78.5 (69.1; 86.2)	41.1 (31.7; 51.1)	55.0 (50.3; 59.6)	67.7 (57.4; 76.5)	59.0 (52.0; 65.8)	.67 (.60; .74)
	ATA-4	44.9 (34.8; 55.3)	78.5 (69.5; 85.9)	65.7 (55.6; 74.5)	60.9 (55.9; 65.6)	62.4 (55.4; 69.1)	.58 (.50; .66)
	BTA-4	56.1 (45.7; 66.1)	64.5 (54.7; 73.5)	59.1 (51.5; 66.4)	61.6 (55.2; 67.6)	60.5 (53.4; 67.2)	.62 (.55; .70)
	EU-4	82.7 (73.7; 89.6)	42.1 (32.6; 52.0)	56.6 (52.1; 61.1)	72.6 (62.0; 81.2)	61.5 (54.4; 68.2)	.63 (.56; .69)
	Kwak-4b	63.3 (52.9; 72.8)	57.0 (47.1; 66.5)	57.4 (50.8; 63.7)	62.9 (55.5; 69.7)	60.0 (53.0; 66.8)	.64 (.57; .71)
	KSThR-4	77.6 (68.0; 85.4)	43.9 (34.3; 53.9)	55.9 (50.9; 60.7)	68.1 (58.3; 76.6)	60.0 (53.0; 66.8)	.63 (.57; .70)

^**ᵻ**^PPV, NPV and DA values were not calculated based on prevalence.

The diagnostic performances at the default setting and Adjusted 1 setting were comparable for all TIRADS whereas Adjusted 2 and Adjusted 3 had lower diagnostic performances. The sole lowering of sensitivity to hyperechoic foci to 3.5 (Adjusted 1), resulted in a slight increase in sensitivity and specificity and a good AUROC (0.71) with ATA TIRADS. The other TIRADS had a slightly lower sensitivity and AUROC with a slight increase in specificity except for KSThR which maintained an AUROC of 0.7. Conversely, the sensitivity of BTA was increased while the specificity was reduced (SEN: 69.4%, SPEC: 58.9%). The AUROC at the adjusted settings was generally lower than at the default setting for all TIRADS.

The Cochran’s Q test indicated differences among the different TIRADS at different settings. Table 3 illustrates these results. At the default setting the most significant differences in sensitivity were between EU and 2 TIRADS (BTA and ATA, p< 0.05) whereas for specificity it was between ACR and KSThR; ACR and BTA and ATA and EU (p< 0.001). The z- test for AUROC paired differences showed the most statistically significant differences between BTA and Kwak, and EU and Kwak (p< 0.001). The AUROC of Kwak was not significantly different from that of ACR (p = 0.289) and ATA (p = 0.795) at the default setting. At all the adjusted settings there were no significant differences between AUROC of different TIRADS (p> 0.05). The most significant difference was between ATA and EU TIRADS at all adjusted settings for both sensitivity and specificity (p< 0.05). EU-TIRADS post-adjustment diagnostic performance measures results were not statistically significant from the default setting results (p> 0.05), except at Adjusted 2. EU and ACR TIRADS sensitivity and specificity had no statistically significant differences in all settings (p> 0.05).

**Table 3:** Pairwise comparison of TIRADS diagnostic performance in CAD-differentiation of malignant and benign thyroid nodules at different settings.

TIRADS Pairs	Performance measures (p-values)											
	Default			Adjusted 1			Adjusted 2			Adjusted 3		
	SEN*	SPEC*	AUROC	SEN*	SPEC*	AUROC	SEN*	SPEC*	AUROC	SEN*	SPEC*	AUROC
**ATA—ACR**	.041	.000	.826	1.000	.000	.262	.003	.000	.546	.000	.000	.079
**ATA—BTA**	1.000	1.000	.158	1.000	.605	.951	.151	.004	.393	.305	.027	.080
**ATA—EU**	.001	.000	.051	.001	.000	.873	.000	.000	.224	.000	.000	.057
**ATA—Kwak**	.539	.054	.795	1.000	.183	.391	1.000	.120	.497	.002	.000	.120
**ATA—KSThR**	.248	.002	.409	1.000	.605	.094	.016	.001	.992	.000	.000	.073
**ACR—BTA**	.001	.000	.019	1.000	.183	.338	1.000	1.000	.814	.000	.000	.427
**ACR—EU**	1.000	1.000	.001	.530	1.000	.268	.289	1.000	.310	1.000	1.000	.377
**ACR—Kwak**	1.000	.108	.289	1.000	.605	.490	.151	.120	.686	.023	.006	.204
**ACR—KSThR**	.013	.000	.248	1.000	.183	.732	1.000	1.000	.554	1.000	1.000	.284
**BTA—EU**	.442	.000	.530	.005*	.046	.860	.007	.238	.091	.000	.000	.865
**BTA—Kwak**	.07*	.018	.000	1.000*	1.000	.486	1.000	1.000	.818	1.000	1.000	.522
**BTA—KSThR**	.206	.206	.168	1.000	1.000	.115	1.000	1.000	.343	.000	.000	.712
**EU—Kwak**	1.000	.054	.000	.040	.183	.472	.000	.011	.159	.001	.013	.694
**EU—KSThR**	1.000	.657	.022	.040	.046	.140	.075	.806	.169	1.000	1.000	.720
**Kwak—KSThR**	1.000	1.000	.030	1.000	1.000	.411	.528	1.000	.604	.047	.053	.816

*adjusted p values with Bonferroni correction for multiple tests comparisons.

### AmCAD-UT sonographic impression of false positive and false negative nodules

**Fig 5:**
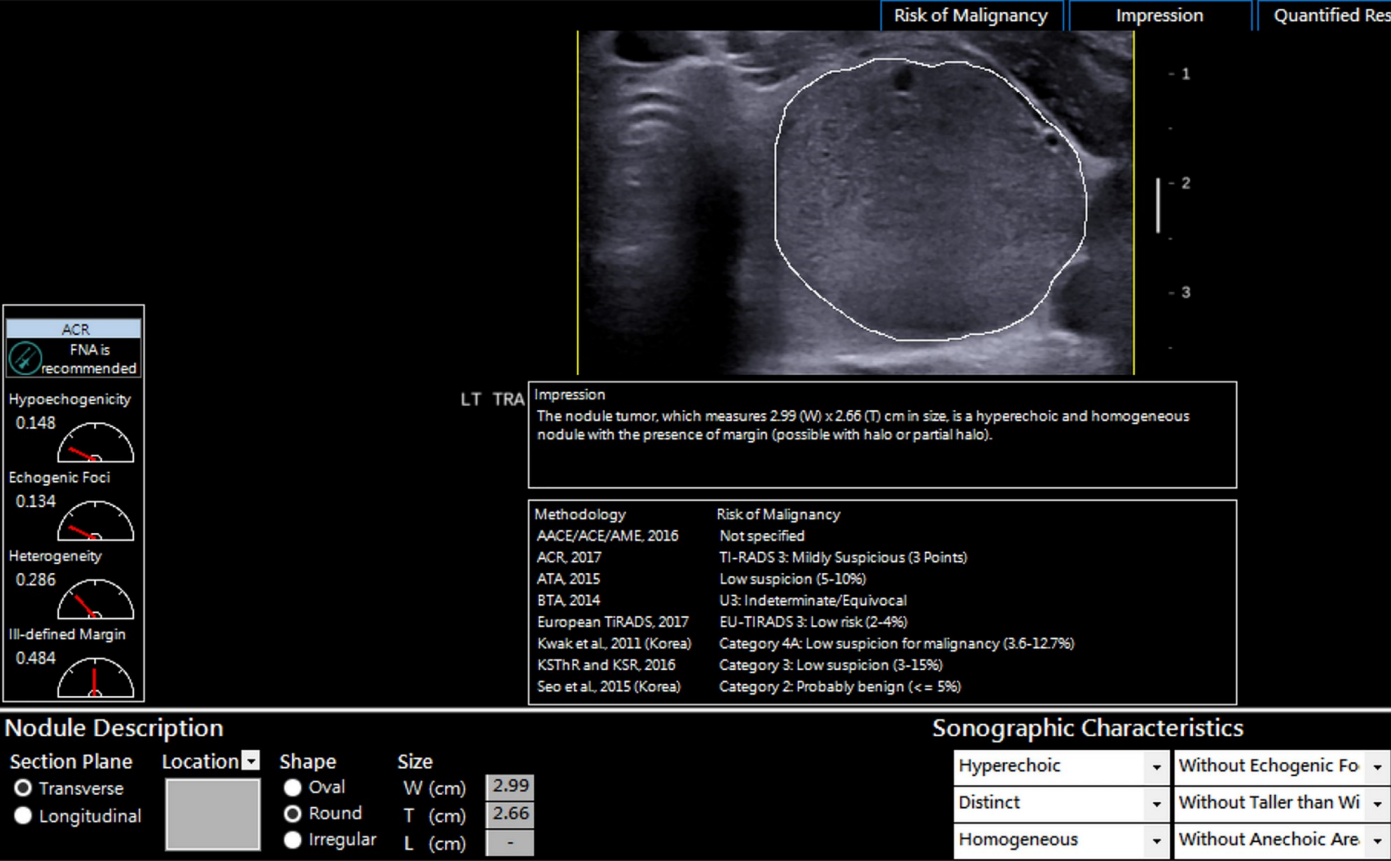
An example of a thyroid nodule misdiagnosed as false negative for malignancy. The misdiagnosis was based on the software’s impression of the hyperechoic and homogenous sonographic features. The histopathological diagnosis of the nodule was papillary thyroid cancer.

**Fig 6:**
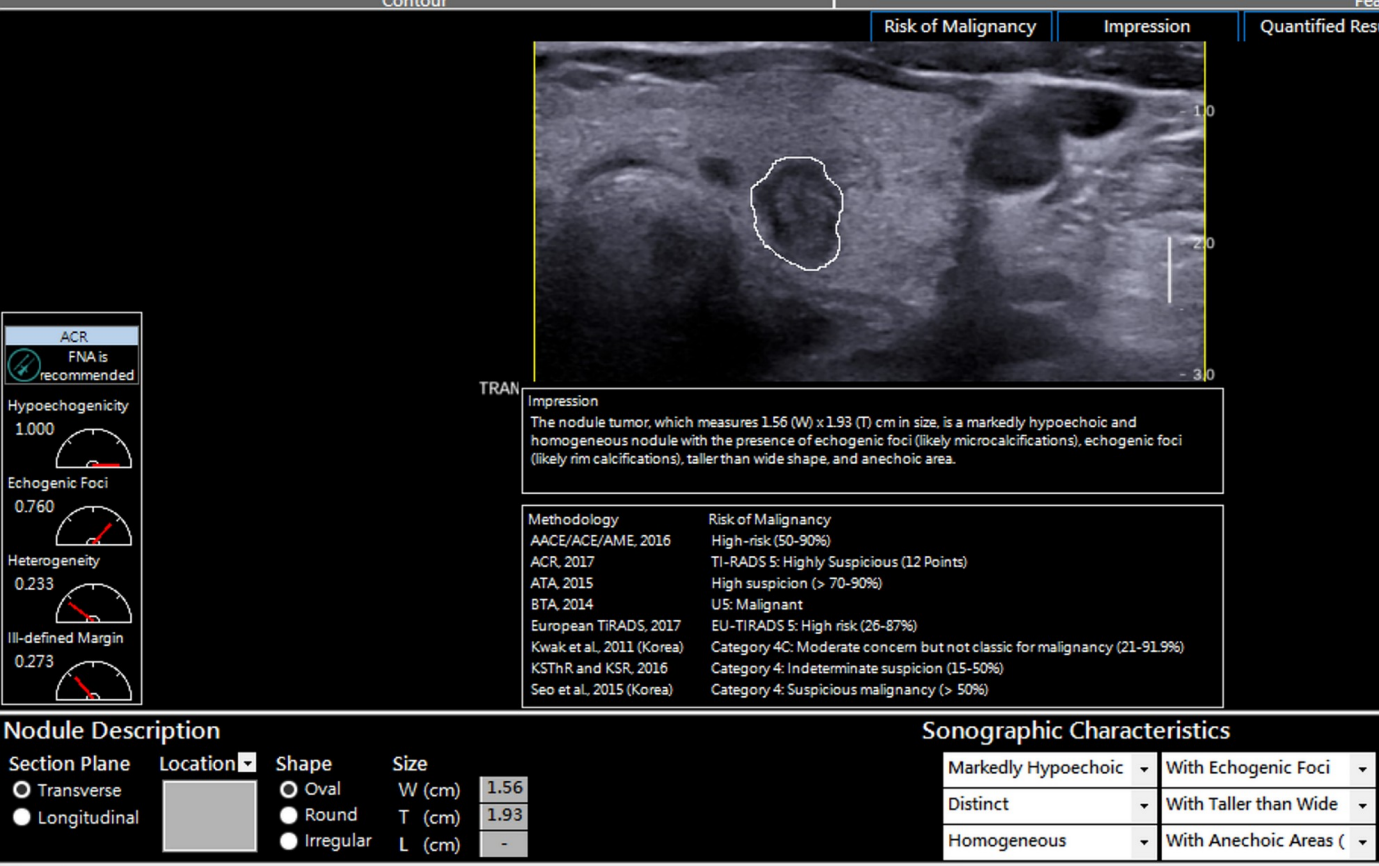
An example of a solid markedly hypoechoic thyroid nodule that was diagnosed as false positive. The nodule was also taller than wide with echogenic foci. The impression of these suspicious sonographic features led to the misdiagnosis by the different TIRADS within the AmCAD-UT software. The nodule was diagnosed as benign with cytology.

There were 48 nodules (32 false positives and 16 false negatives) that were consistently misdiagnosed across all TIRADS at the different AmCAD-UT settings. Based on the software’s sonographic impression output, 75% (24/32) of the false-positive nodules were predominantly solid, 53% (17/32) had irregular margins and 78% (25/32) had a homogenous echotexture. 46% (6/13) of false-positive nodules with detected echogenic foci were attributed to mixed calcifications, while 59% (19/32) of all false-positive nodules had no calcifications. Echogenicity distribution varied, with a combined 44% total (7/32 and 7/32) for hypoechoic and markedly hypoechoic. All 16 false-negative nodules had regular margins 75% (12/16) were heterogeneous, 56% (9/16) were hyperechoic and 38% (6/16) had microcalcifications. Typical examples of nodules that the CAD misdiagnosed based on the impression of sonographic features are shown in Figs 5 and 6.

## Discussion

CAD approaches have been found to be more objective and to perform comparatively accurate to expert human assessment of ultrasound features. This study sought to evaluate the diagnostic performance of adjusted settings of AmCAD-UT in comparison with the default setting for thyroid nodule differentiation based on six TIRADS. The diagnostic performance of AmCAD-UT was higher at the default setting than at different adjusted settings. EU TIRADS had the highest sensitivity (82.7%); ATA the highest specificity (66.4%) while ATA, ACR, KSThR and Kwak all had good AUROC with Kwak being the highest (0.74). Human subjective interpretation studies have established the high sensitivity and NPV of EU TIRADS; a high AUROC with Kwak TIRADS and comparable diagnostic efficiency among ACR, ATA, Kwak and KSThR TIRADS with high sensitivities [23–26]. In CAD assessment, Reverter et al. [16] evaluated the performance of AmCAD-UT at default settings using ATA, EU and AACE/ACE/AME TIRADS. In that study, ATA TIRADS overall had the highest diagnostic performance and a sensitivity comparable to that of an expert using ATA TIRADS. Contrarily, at the default setting, this present study had a lower sensitivity for ATA than that study (SEN: 69.4% vs 87%). However, the ATA specificity was comparable (SPEC: 66.4% vs 68.8%) as well as the EU TIRADS sensitivity and specificity (SEN: 82.7% vs 85.2%; SPEC: 42.1% vs 50.2%). The differences in ATA sensitivity results may be attributed to the differences in the TIRADS cut-off category criteria, study design and study populations.

In this present CAD study, the Adjusted 1 setting had comparable results with the default setting; however, with a slight improvement in specificity and sensitivity for ATA TIRADS and a minimal increase in specificity for most TIRADS. The differences in performance with the adjustment of settings may be explained by the difference in malignancy risk stratification criteria for the different TIRADS based on pattern-based approaches (ATA, EU, BTA and KSThR) or score-based approaches (ACR and Kwak) [23, 27, 28]. Furthermore, there are inconsistencies mainly in the categorisation of echogenic foci and echogenicity among the different TIRADS [29]. While ATA may fail to classify nodules with mixed calcifications, Kwak will interpret them as having microcalcifications thereby resulting in a higher fitted malignancy probability for calcifications [30, 31]. Sole reduction of the sensitivity detection of hyperechoic foci likely hindered the detection of subtle calcifications for the malignancy risk computation thereby slightly lowering the overall sensitivity while improving specificity. This suggests that AmCAD-UT sensitivity detection adjustments are most advisable for the individual analysis of problematic suspicious sonographic features that affect the malignancy risk estimation based on the TIRADS choice. An example is the adjustment of hyperechoic foci and anechoic areas settings separately for a hypoechoic nodule with mixed echogenic foci without other suggestive features or corresponding clinical history. The focus on calcifications and hypoechogenicity features separately could help ascertain the extent each feature influences the CAD output based on the different TIRADS classification disparities.

Although the current study did not involve multiple users, the sensitivity at the default and Adjusted 1 setting using Kwak, KSThR, and ATA TIRADS were comparable to a previous CAD study’s findings for less experienced ultrasound users, while EU TIRADS had higher sensitivity (82.7%) than that same study which yielded an average sensitivity of about 72% [32]. For the same TIRADS category, our study had similar sensitivity (79.6%) to that of an ACR- based CAD development study for sole CAD and a junior radiologist using CAD (80.6% and 78.1%, respectively); although that study had a higher diagnostic performance for all other measures [33]. Similarly, at the default setting, the AUROC of above 0.70 with Kwak, KSThR, ACR and ATA TIRADS in our study, corresponded with that of a recent multicentre and multi-reader AmCAD-UT study which demonstrated an average of 0.792 AUROC regardless of user experience [34]. However, the multi-reader study outcomes were not stated as specific to any TIRADS. Due to the limited evaluation of CAD diagnostic performance using multiple TIRADS and readers, future studies are warranted to verify the influence of the CAD user experience based on different TIRADS and settings.

AmCAD-UT ultrasound feature impression analysis in this current study showed that most of the misdiagnosed nodules had some typical features of suspicion for malignancy or benignity. The interpretations of solid, homogenous nodules with irregular margins and/or echogenic foci features (such as the presence of colloid) were likely the key contributors to the false-positive diagnosis of some benign nodules. This can be attributed to the high thyroid malignancy prediction in the presence of multiple suspicious features established in several non-CAD studies [35–37]. Furthermore, the presence of punctate echogenic foci with a comet-tail artefact in a hypoechoic solid nodule and the presence of multiple calcifications can result in a high malignancy rate and PPV (77.8% and 96% respectively) [38]. This may account for the misdiagnosis of benign nodules interpreted as having mixed calcifications in the present CAD study. The TIRADS category 4 cut-off criteria, likewise, contributed to the misdiagnosis findings because in some TIRADS it denotes intermediate suspicion which presents diagnostic challenges even with human assessment. These misdiagnoses confirm the need for clinical correlation for accurate diagnosis even with the complementary use of thyroid CAD for diagnosis.

This study had several limitations. Due to its retrospective nature, some histopathological diagnosis data of some nodules were not available which prevented the analysis of pathological factors. Furthermore, selection bias cannot be excluded due to the selection of patients’ images with FNAC and/or histopathology results as opposed to data from the general population. The study design was not typical of a hospital setting whereby CAD results complement those acquired by a clinician since only one user conducted the CAD analysis thereby hindering inter-rater agreement analysis. However, this is a first study to compare the sole diagnostic performance of AmCAD-UT at different adjusted settings and the study findings may help guide future studies with multiple CAD users. Future standardized prospective studies with larger sample sizes and comparative approaches may be useful in increasing the validity of the findings and improving generalizability.

## Conclusion

Based on this study, the diagnostic performance of AmCAD-UT was best for all 6 TIRADS at the default setting. The default setting was best for maximising sensitivity for all TIRADS, with EU-TIRADS having the highest sensitivity. However, there may be potential for improved specificity without compromising the sensitivity at a hyperechoic foci detection setting of 3.5 with other settings maintained at median values. Further large prospective studies are warranted to validate these findings.
